# Dual-Energy X-Ray Absorptiometry (DEXA) Scan Versus Computed Tomography for Bone Density Assessment

**DOI:** 10.7759/cureus.13261

**Published:** 2021-02-10

**Authors:** Malak Alawi, Azra Begum, Mohammed Harraz, Hani Alawi, Shahd Bamagos, Abdulmalek Yaghmour, Lubna Hafiz

**Affiliations:** 1 Radiodiagnosis, King Abdul Aziz Hospital, Makkah, SAU; 2 Radiodiagnosis, Mansoura University Hospital, Mansoura, EGY; 3 Family Medicine, Primary Health Center Clinic, Makkah, SAU; 4 Radiodiagnosis, Umm Al Qura University, Makkah, SAU; 5 Family Medicine, Al Zaher Primary Health Care Center, Makkah, SAU

**Keywords:** dexa, ct, bone density, t-score, bmd, bone mineral density, correlation, osteoporosis, osteopenia, who

## Abstract

Rationale and objective

Osteoporosis, a common non-pathological disease of bones, has been the cause of many disastrous consequences, in terms of physical, psychological, social, and economic loss. Therefore, it is crucial to diagnose it early for timely prevention and treatment of osteoporotic fractures. Dual-Energy X-Ray Absorptiometry (DEXA) is currently routinely used for determining bone mineral density. However, it has its limitations. Nowadays, CT technology has advanced so rapidly that the Hounsfield units (HU) values can be used in opportunistic screening for osteoporosis in patients during routine CT abdomen for other causes. Hence, there would be no need for additional study with DEXA and also reduce radiation exposure. The aim of our research is to determine whether there is a correlation between the bone mineral density and the T-score measured by DEXA and the HU values measured from the diagnostic CT images of L1-4 vertebrae. Also, to determine reference CT values that would help in screening the patients with osteoporosis.

Materials and methods

We conducted a retrospective study of 78 female patients who underwent CT lumbar spine, abdomen, and pelvis in our hospital between the years 2016-2020. We collected data of patients who performed DEXA and CT scans within an interval of up to two years. The final collected data was analyzed to find correlation values of HU with age group and with DEXA bone mineral density (BMD) and T-score using Pearson correlation coefficient.

Results

The mean of the 78 patients was 61.1 (range 37-88 years). Mean HU values decreased consistently with age, from 202.17 HU in the fifth decade to 71 HU in the ninth decade. Average L1-4 HU values ranged from 71 HU to 202.17 HU (mean with standard deviation), while their T-score ranged from -4.4 to +2.4 (mean was -1.7±1.41), and their BMD ranged from 0.62 to 1.465 g/cm^2 ^(mean, 0.974±0.175 g/cm^2^). For each lumbar vertebra, the correlations of HU values with bone mineral density and T-score were calculated separately. For L1-4 vertebrae, the correlation coefficients (r^2^) for the HU value and T-score were 0.544, 0.600, 0.611, and 0.600, respectively. The correlation coefficients (r^2^) for the HU value and bone mineral density were 0.581, 0.623, 0.653,0.612, respectively. All the calculated correlations were significant (p<0.001). Therefore, it was concluded that there was a positive correlation between the HU values and the DEXA for the BMD and between the HU values and the T-score. Based on the WHO guidelines, the T-scores of the lumbar vertebrae were classified into three groups. The mean HU values for the subjects in the normal group were 174.05 (95% confidence interval, 153-194.49), in the osteopenia group were 120.45 HU (95% confidence interval, 106.98-133.91), and in the osteoporosis group were 115 HU (95% confidence interval, 104.60-125.40). The differences in the mean HU values between the groups were significant.

Conclusion

On analyzing the results of our study, we reached the conclusion that there is a positive correlation between the HU calculated from CT with automated exposure control and BMD calculated from the DEXA. Thus CT scans done for various reasons, for example, the abdomen, lumbar spine, etc. can provide us with information about the patient’s bone density as well. CT is a very popular, easily accessible, reproducible, and reliable tool for measuring HU values and thereby in the opportunistic screening of osteoporosis.

## Introduction

Osteoporosis is a disease of the bones in which low bone mass and structural deterioration of bone tissue lead to an increase in bone fragility. It is by far the most common metabolic bone disease which has affected over 200 million people all over the world. It has also been the cause of many disastrous consequences, in terms of physical, psychological, social, and economic loss. The unfortunate reason for this is that it is often overlooked and under-treated because it does not show any clinical symptoms until it suddenly manifests as a fracture. Therefore it is very crucial to diagnose it early for timely prevention and treatment of and osteoporotic fractures and their unfortunate consequences [1,2,3].

Osteoporosis is monitored using the measure of bone mineral density (BMD) as it is directly related to bone strength [4]. BMD measurements are classified according to the T-score as specified by the World Health Organization (WHO) in 1994 [5]. A T-score is the standard deviation of the BMD of an individual patient compared with a young, healthy reference population, matched for sex and ethnicity. A T-score that lies between the values of -2.5 and -1 is defined as osteopenia while a T-score that is equal to or less than -2.5 is defined as osteoporosis. Initially, this definition was intended to be used in postmenopausal women. Later, the International Society for Clinical Densitometry (ISCD) modified and used this definition to classify BMD in pre-and post-menopausal women, men, and children by using Dual-Energy X-ray Absorptiometry (DEXA), which calculated the BMD (grams per square centimeter) as well as the Z-scores and T-scores. Z-scores are standard deviations compared with an age-matched reference population, while T-scores are standard deviations compared with a young adult reference population [6].

Dual-energy X-ray Absorptiometry (DEXA) and quantitative computed tomography (QCT) of the lumbar spine were considered as preferred methods for the evaluation of BMD. DEXA was recommended by the World Health Organization (WHO) as a gold standard for diagnosing osteoporosis. Because of its availability, relatively minimal radiation exposure, and simplicity of use, DEXA is the most commonly employed quantitative radiologic method to assess bone mass [7].

However, DEXA also has disadvantages that need to be kept in mind:

1) It is a two-dimensional measurement, which only measures density/area (in grams per square centimeter) and not the volumetric density (in milligrams per cubic centimeter) such as with quantitative computed tomography (CT).

2) Areal BMD is susceptible to bone size and will thus overestimate fracture risk in individuals with a small body frame, who will have lower areal BMD than normal-sized individuals. 

3) Spine and hip DEXA are also sensitive to degenerative changes, and individuals with substantial degenerative disease will have increased areal density, which will suggest a lower fracture risk than is actually present. All structures overlying the spine, such as aortic calcifications, or morphologic abnormalities, such as after laminectomy at the spine, will affect BMD measurements; it is also critical to check DEXA images for artifacts, which may alter BMD values [6].

Another tool to measure BMD is using quantitative computed tomography (QCT). This idea was suggested in the 1970s [7]. In the initial days, CT technology was not very well developed as it took a long time and there was more exposure to radiation. Therefore, it was not considered. However, now CT technology has made rapid advancement, making it a promising tool in measuring BMD [8].

The Hounsfield Unit (HU) values can be a very useful index to opportunistically screen patients who undergo routine CT abdomen and pelvis for other reasons and thus obviate the need to do another test like DEXA for BMD assessment. There will also be a reduction in radiation dose.

Aims and objectives

Our study was aimed at finding out whether there was a correlation between the bone mineral density and the T-scores measured by DEXA and the HU values measured from the diagnostic CT images of L1-L4 vertebrae. We also aimed to determine reference CT values that would help in screening patients with osteoporosis.

## Materials and methods

We conducted a retrospective study of 78 female patients, of both pre- and postmenopausal age groups and irrespective of risk factor status, who underwent CT lumbar spine, abdomen, and pelvis in our hospital between the years 2016-2020. Data of patients who underwent DEXA and CT scans within an interval of up to two years was collected. The patients with extensive spine degenerative changes or post-surgical spine intervention, etc., were excluded from our study. The final collected data was analyzed to find correlation values of HU with age group and with DEXA bone mineral density and T-score using Pearson correlation coefficient.

All imaging was performed by a 64-slice multidetector CT (MDCT) scanner (Discover, GE Healthcare, Chicago USA). The CT parameters included a slice thickness of 1.0 mm, a tube voltage of 120 kV, a tube current of 330 mA (DoseRight automated exposure control system), and a bone reconstruction algorithm (window width/window level, 2050/250). Two-dimensional reconstructions were obtained in the coronal and sagittal planes.

DEXA scans were performed with the use of a Lunar Bone Densitometer (GE Healthcare). The T-scores and the bone mineral density (grams per square centimeter) were obtained from the DEXA scan for the L1 to L4 lumbar vertebrae.

**Figure 1:**
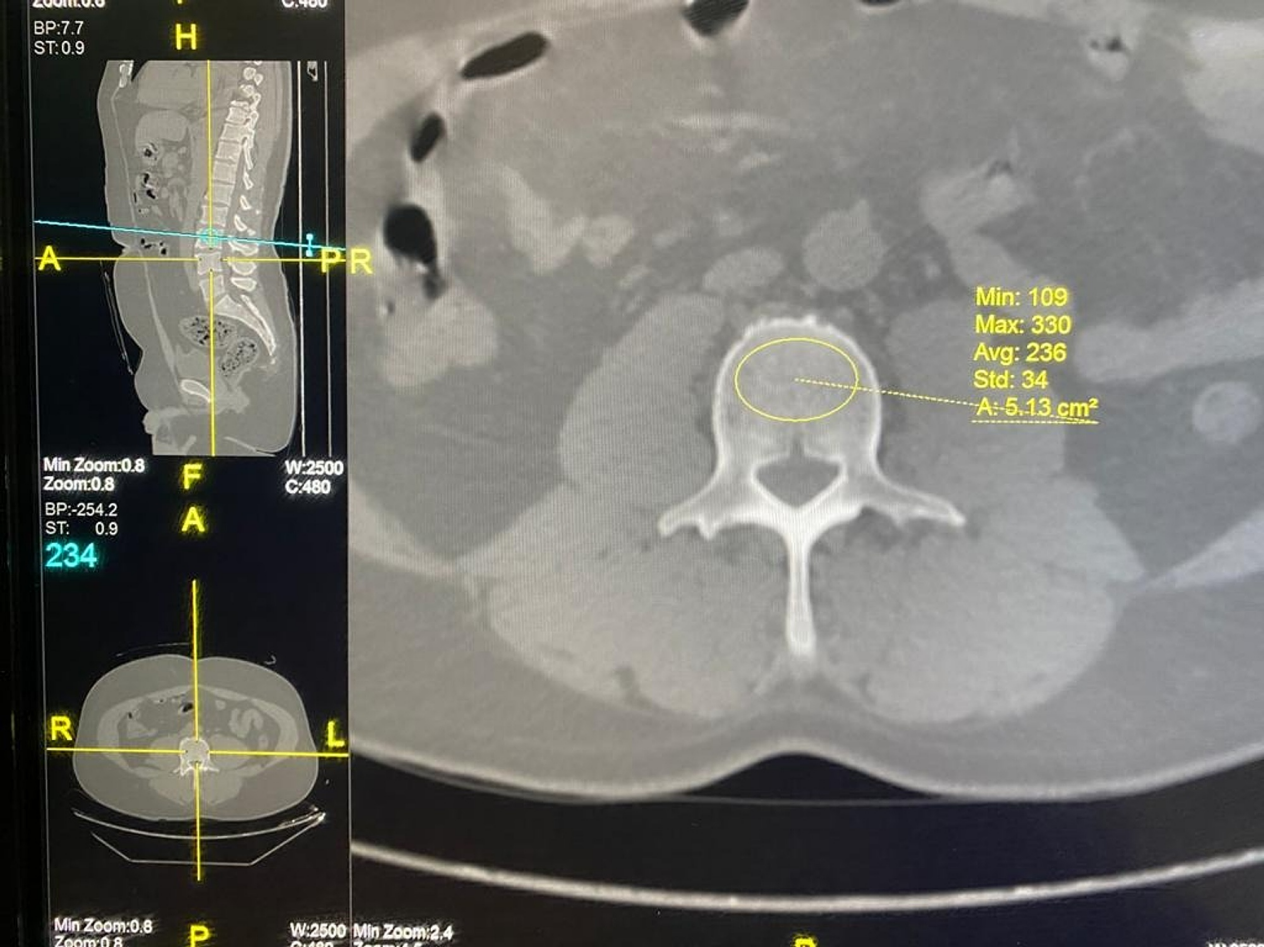
Method to measure HU value on CT image for each vertebra Axial CT images of lumbar vertebrae illustrating the method of determining HU values by using the PACS system. Multi-planar reconstructed images (A) an oval region of interest (ROI) is placed over the axial plane in the mid vertebra level. PACS: picture archiving and communication system

The HU values were calculated by using a picture archiving and communication system (PACS; GE Digital Healthcare). An elliptical region of interest was placed over the axial CT image of the lumbar vertebrae (L1 till L4) excluding the cortices. A protocol described by Pickhardt et al. [9], was used to measure the HU for each vertebra (Figure 1).

Statistical analysis

The Statistical Analysis was carried out using Statistical Package for Social Sciences (SPSS) software, Version 22.0 (IBM Corp., Armonk, NY, USA). The correlation parameters between DEXA and CT were calculated for HU values of each vertebral level, L1 till L4. All statistical tests were two-sided and performed at a significant level of p<0.05. Correlation values of HU with age group and with DEXA bone mineral density and T-score using Pearson correlation coefficient (0.0-0.19 -very weak correlation, 0.20-0.39 - weak correlation, 0.40-0.59 - moderate correlation, 0.60-0.79 - strong correlation, 0.80-1.0 - very strong correlation). Mean and 95% confidence intervals were calculated of patients with bone density divided into three groups - normal, osteopenia, and osteoporosis - as per WHO guidelines.

## Results

**Figure 2:**
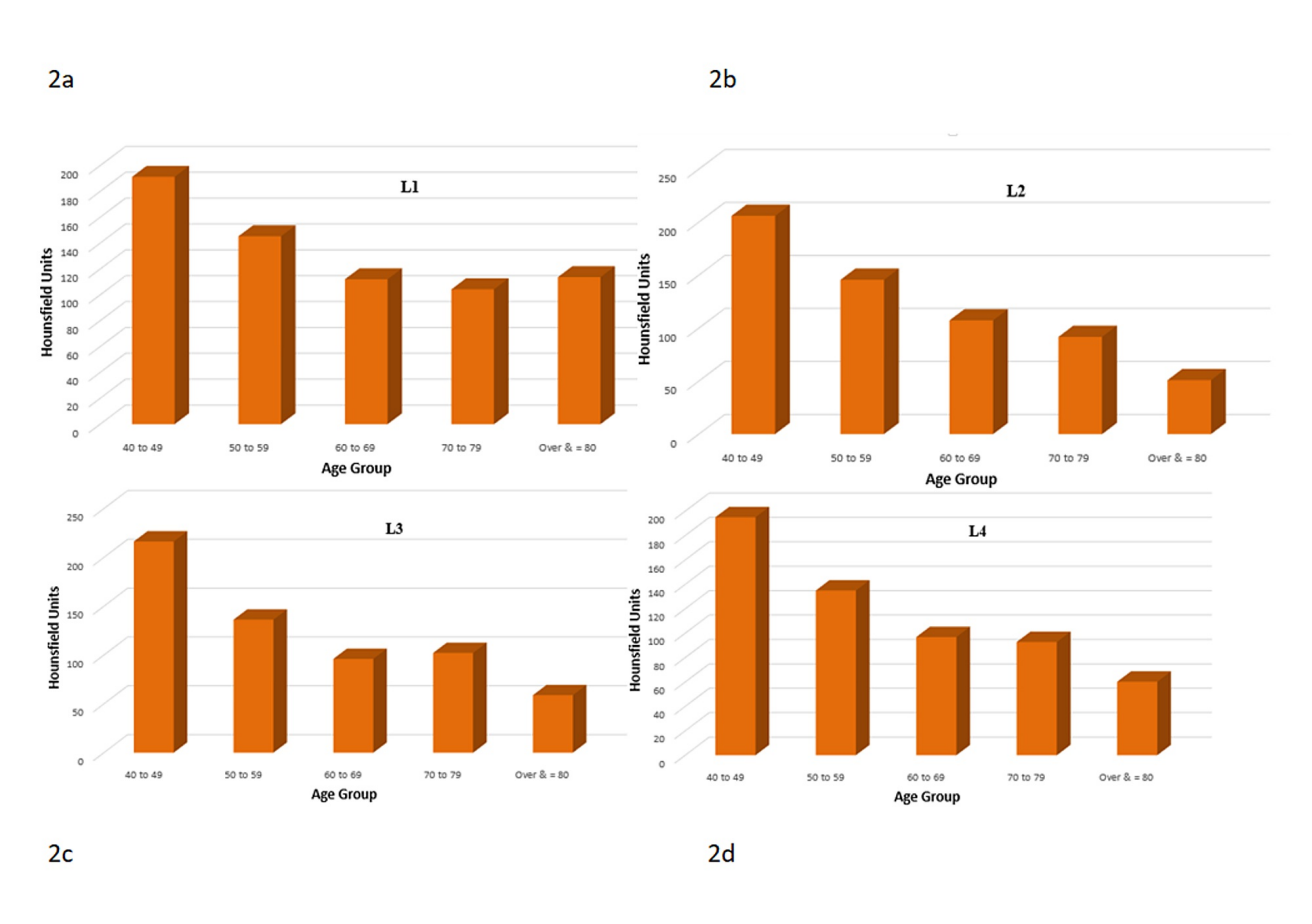
Hounsfield Unit values on CT of lumbar vertebrae versus age Mean Hounsfield unit values among decadal age groups decreases as age increases. 2a - L1 vertebra HU values versus age groups; 2b - L2 vertebra HU values versus age groups; 2c - L3 vertebra HU values versus age groups; 2d - L4 vertebra HU values versus age groups.

The mean age of the 78 patients was 61.1 years (range 37-88 years). It was observed that the mean HU values showed a tendency to decrease with age (Table 1, Figure 2).

**Table 1:** Hounsfield Unit values of lumbar vertebrae versus age Data of Hounsfield unit values obtained from lumbar computed tomography scans arranged according to decades of life.

Age (Years)	Patient No.	Hounsfield Units (Means ± Standard Deviation)				Average
		L1	L2	L3	L4	
40 to 49	3	191.3333 ± 21.45538	206 ± 4.358899	216.3333 ± 31.65965	195 ± 16.52271	202.1667 ± 3.826988
50 to 59	35	145.4286 ± 41.12555	145.6857 ± 45.01031	136.3429 ± 48.57374	135 ± 49.87101	140.6143 ± 42.37192
60 to 69	18	112.1111 ± 31.32165	107.3333 ± 28.77090	96.11111 ± 28.51189	96.8333 ± 24.39684	103.0972 ± 25.90553
70 to 79	14	104.3571 ± 28.78997	91.78571 ± 30.40035	102.2143 ± 34.53466	92.78571 ± 26.46094	97.78571 ± 24.23282
Over & = 80	4	113.75 ± 46.28445	51 ± 24.38579	59 ± 36.1755	60.25 ± 51.93826	71 ± 29.62333

**Figure 3:**
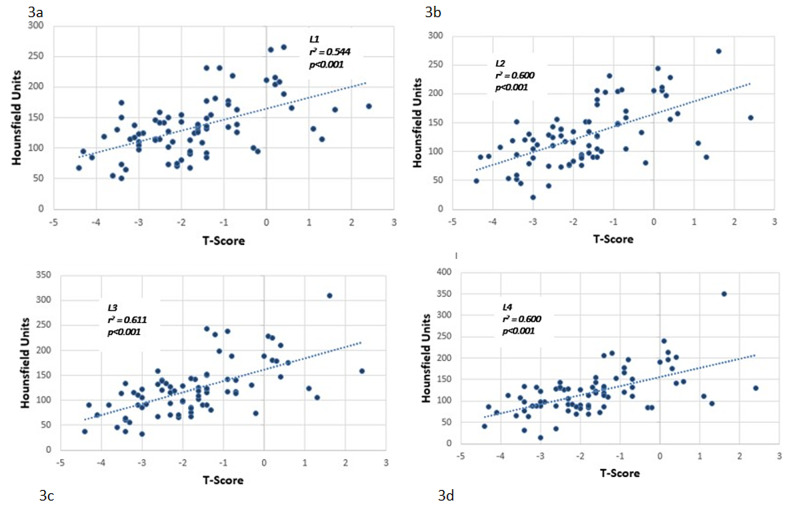
Scatter plot showing the correlation between Hounsfield unit values from CT and T-scores form Dual-Energy X-Ray Absorptiometry Scatter plot showing correlations between Hounsfield unit values obtained from CT and T-scores obtained from Dual-Energy X-Ray Absorpti­ometry for lumbar vertebrae: 3a - L1, 3b - L2, 3c - L3, 3d - L4. It showed significant correlation coefficients (p<0.001).

**Figure 4:**
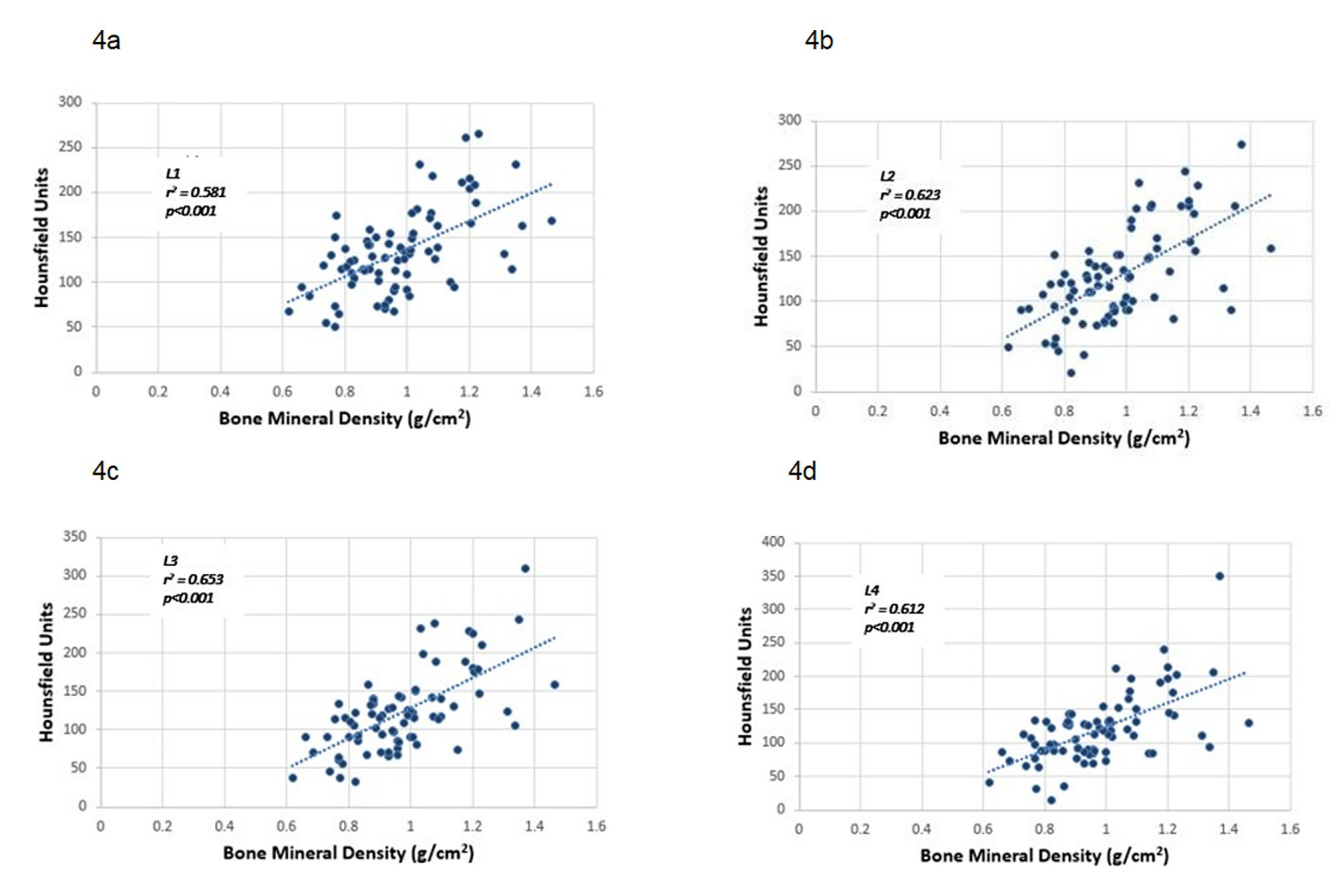
Scatter plots showing the correlation between Hounsfield unit values obtained from CT scans and bone mineral density obtained from Dual-Energy X-Ray Absorptiometry Scatter plots showing correlations between Hounsfield Unit values obtained from CT scans and bone mineral density obtained from Dual-Ener­gy X-Ray Absorptiometry for lumbar vertebrae: 4a - L1, 4b - L2, 4c - L3, 4d -L4. It showed significant correlation coefficients (p<0.001).

Average L1-4 HU values ranged from 71 HU to 202.17 HU (mean with standard deviation), while their T-score ranged from -4.4 to +2.4 (mean was -1.7±1.41), and their bone mineral density ranged from 0.62 to 1.465 g/cm2 (mean 0.974±0.175 g/cm2). For each lumbar vertebra, the correlations of HU values with bone mineral density and T-score were calculated separately. For L1-4 vertebrae, the correlation coefficients (r^2^) for the HU value and T-score were 0.544, 0.600, 0.611, and 0.600, respectively (Figure 3). The correlation coefficients (r^2^) for the HU value and bone mineral density were 0.581, 0.623, 0.653, and 0.612, respectively (Figure 4). All the calculated correlations were significant (p<0.001). Therefore, it was concluded that there was a positive correlation between the HU values and the DEXA for the BMD and between the HU values and the T-score.

According to the WHO guidelines, the T-scores of the lumbar vertebrae were classified into normal (-1.0 or greater), osteopenia (less than -1.0 and greater than -2.5), and osteoporosis (-2.5 or less). The mean HU values for the subjects in the normal group were 174.05 (95% confidence interval, 153-194.49), in the osteopenia group were 120.45 HU (95% confidence interval, 106.98 - 133.91), and in the osteoporosis group were 115 HU (95% confidence interval, 104.60-125.40) (Table 2). There was no significant difference in the mean HU between the osteopenia and osteoporosis groups. However, the difference in the mean HU values was significant between the normal group and osteopenia (p<0.05).

**Table 2:** Mean and 95% confidence intervals of patients with bone density divided into three groups-normal, osteopenia and osteoporosis

	T-Score	Hounsfield Units	
		Mean and SD	95% Confidence Interval
Normal	≥-1.0	174.057 ± 46.08	153.63 – 194.49
Osteopenia	-1.0	120.45 ± 37.34	106.98 – 133.91
Osteoporotic	-2.5≤	115 ± 41.30	104.60 - 125.40

## Discussion

On observation and analysis of the data, it was seen that there is a moderate correlation between the HU values obtained from CT and the bone mineral density and T-score determined by DEXA. This could probably be due to the fact that DEXA scan calculations include the cortex and the posterior elements, while only the trabecular portion of the vertebra was used to determine the HU value on CT. 

There are a few studies which have shown the possibility of using diagnostic CT images (with automated exposure control) for estimating bone mineral density [10-13].

DEXA is currently routinely used for determining bone mineral density, because of the advantage that it is easily available, easy to perform, and has a low radiation dose (0.009-0.027 mSv). While radiation dose of Computed Tomography (CT) is higher (0.06-2.5 mSv) [14].

Previously, quantitative CT required the use of a calibration phantom with a known density. The phantom was scanned with the patient to convert HU values into bone mineral density and to permit calibration of other factors that may interfere with the results [7]. With the advent of automated exposure control on modern CT scanners, the calibrating phantoms that were historically used for quantitative CT are not required [10].

Our study showed that there was a moderate correlation between HU values and T-score. Therefore, patients who have decreased bone density can be identified easily during routine CT scans done for various reasons and directed to DEXA imaging, which can help to determine the category of bone density - normal, osteopenia, or osteoporosis. Thus, CT can be used as an opportunistic screening tool for decreased bone density.

The results of our study were similar to the previous studies which support the use of CT in screening patients for decreased bone density. We suggest a reference value of 150 HU on CT imaging as a cut-off for determining patients with low bone density. Our reference value is corroborating with the previous studies [9,15,16].

As stated earlier, the radiation dose of CT is higher than DEXA. Therefore, though CT may not be recommended as the standard for bone density measurement, it can still be used as an opportunistic tool in patients undergoing routine CT scans for other reasons to detect those with low bone density and who can be referred for DEXA imaging and further treatment.

The limitations of our study are that our measurements and calculations were done on a single type of CT scanner and DEXA machine. We do not know the reproducibility of the results in other different types of CT and DEXA scanners. Another limitation is that the number of patients was limited and hence the need for a large population to determine the accuracy and reliability of the results. The third limitation was the gap of one to two years between CT and DEXA imaging may alter the results. Also, the HU values were calculated from trabeculated bone on CT, while DEXA values are calculated including the cortical bone with the trabeculated bone, which can cause a difference in correlation of both CT and DEXA.

## Conclusions

On analyzing the results of our study, we reached the conclusion that there is a positive correlation between the HU calculated from CT with automated exposure control and BMD calculated from the DEXA. The determination of reference cut off value of HU can be applied in all routine CT scans for detecting patients with low bone density. Thus, CT being a very popular, easily accessible, reproducible, and reliable tool for measuring HU values, can thereby be useful in the opportunistic screening of patients with decreased bone density, who can be referred for DEXA and subsequent management.
